# Lack of infrastructure, social and cultural factors limit physical activity among patients with type 2 diabetes in rural Sri Lanka, a qualitative study

**DOI:** 10.1371/journal.pone.0192679

**Published:** 2018-02-20

**Authors:** Arjuna Medagama, Manoj Galgomuwa

**Affiliations:** Department of Medicine, Faculty of Medicine, University of Peradeniya, Peradeniya, Sri Lanka; Florida International University Herbert Wertheim College of Medicine, UNITED STATES

## Abstract

**Introduction:**

South Asians have high prevalence of diabetes, increased cardiovascular risk and low levels of physical activity (PA). Reasons for low levels of PA have not previously been explored among Asians living within their endogenous environment. This qualitative study was performed to explore the contextual reasons that limited PA among type 2 diabetic patients living in a rural community.

**Methods:**

Purposeful sampling recruited 40 participants with long standing type 2 diabetes for this qualitative study. Semi-structered questions utilising in-depth interviews were used to collect data on PA patterns, barriers to PA and factors that would facilitate PA. The interviews were digitally recorded and transcribed. Data were analyzed using a framework approach.

**Results:**

The sample consisted of 11 males and 29 females. Mean age was 55.4 (SD 8.9) years. The mean duration of diabetes in the study population was 8.5 (SD 6.8) years. Inability to differentitate household and daily activities from PA emerged as a recurring theme. Most did not have a clear understanding of the type or duration of PA that they should perform. Health related issues, lifestyle and time management, envronmental and social factors like social embarrassment, prioritizing household activities over PA were important factors that limited PA. Most stated that the concept of exercising was alien to their culture and lifestyle.

**Conclusion:**

Culturally appropriate programmes that strengthen health education and empower communities to overcome socio-economic barriers that limit PA should be implemented to better manage diabetes among rural Sri Lankan diabetic patients.

## Background

Sri Lanka is a developing country with a population of 21 million inhabitants and a rapidly increasing burden of non-communicable diseases (NCD) [1].

In 2005, the prevalence of hypertension, diabetes and dysglycaemia was 20%, 11% and 20% respectively [2]. Sri Lanka is a country with very high mortality from cardiovascular disease [3]. Prevalence of diabetes in Sri Lanka, which was around 2.0% in the early nineties has increased by about five-fold during the last two decades [4,5].

Diet and physical activity (PA) are two important modifiable risk factors that play an important role in the incidence, management and outcomes of diabetes [6]. The American Diabetes Association (ADA) recommends two types of PA for individuals with diabetes, which includes aerobic exercises and strengthening exercises. The ADA recommends 30 min of moderate to vigorous intensity aerobic exercise for at least 5 days a week or up to a total of 150 min per week, and resistance training of some type at least two times per week in addition to aerobic activity [7].

Physical inactivity is identified as the fourth leading risk factor for overall mortality globally [8]. Sedentary living is responsible for one third of deaths due to Coronary Heart Disease (CHD) and diabetes, diseases for which physical inactivity is a risk factor [9]. PA is recognized to increase insulin sensitivity, reduce cardio-vascular risk factors, reduce mortality and improve quality of life [10].

Eighty percent of Sri Lankans still dwell rurally. Although, health and education indices are more in comparison with the western world, infrastructure development has not kept pace with global trends [11].

The typical Sri Lankan village consists of a self-contained microenvironment with its own agricultural land, housing, religious, schooling and sometimes basic medical amenities. However, with improving socio-economic conditions the villages tend to be less isolated with a mixture of more urban customs and practices.

Although Sri Lanka harbours a large population of patients with diabetes, very little is known about PA patterns of its inhabitants. A national study revealed that over 60% all Sri Lankan adults report being highly physically active[8]. Very little data exists on PA patterns of Sri Lankan adult diabetic patients. A subset analysis of a national study revealed 13.9% of diabetic individuals to be inactive using the short version of the IPAQ[12]. However, the reason leading to inactivity of has not been examined.

The aim of the current study was to determine barriers to physical activity among a group of patients with type 2 diabetes attending a large multi-ethnic tertiary care diabetes facility in Sri Lanka. The study also explored the associations between physical inactivity and socio-demographic characteristics.

## Methods

Institutional ethical clearance was obtained from the Institutional Ethics Review Committee (IERC) of the Faculty of Medicine, University of Peradeniya, Sri Lanka. All participants gave informed written consent. The study was performed at the diabetes facility at Teaching Hospital Peradeniya, Sri Lanka from 2nd February 2015 to 26^th^ August 2015. Teaching Hospital Peradeniya, located on the outskirts of a major city, serves a catchment population of semi-urban and rural dwellers.

A purposeful sample was drawn from a larger study, evaluating the PA of adult patients with type 2 diabetes. Four hundred patients were recruited in to the larger study, from which 45 patients were selected. The selection was done ensuring male and female representation to be the same and proportionate numbers were selected from the each age category (less than 40 years, 40–60 years and more than 60 years). The patients attend this clinic routinely for their monthly supply of medicines and routine consultation with a physician. None of the recruited patients had any acute illnesses necessitating an out of routine consultation or hospital admission during the past 2 months. The 45 patients thus selected were approached by a trained Research Assistant (RA) on their regular clinic day and were given an information sheet and verbal clarification regarding the study. Male and female patients who were between 18–70 years were recruited and pregnant females were excluded from the study. Patients consenting to be included in the study were then given a date for an interview and they had the freedom to withdraw from the study. The interview was typically was within 2 months of the intital recruitment date. In depth interviews were selected over focus group discussions in this community due to social and cultural reasons.

The clinical team looking after the patient was intentionally kept out of the data collection, as this may have influenced the answers provided by the subjects.

Each scheduled interview lasted an average of 30 minutes and was undertaken at the Diabetes Clinic at the Teaching Hospital, Peradeniya. All interviews were conducted by one RA using a topic guide, which asked about the patients’ type and duration of PA, reasons for not engaging in PA and barriers to initiating and maintaining an execise schedule. The interviews were conducted using open-ended, semistrctured questions to guide the particpants and to maintain uniformity between all the interviews. The language of the interviews was Sinhales as all the participants were fluent in this language. All patients were informed that their interviews will be recorded, transcribed and analyzed while maintaining confidentiality.

The data collected included socio-demographic details of age, gender, marital status, occupation, income, area of residence, anthropometric and diabetes related data.

### Data handling and analysis

The data were initially transcribed in Sinhalese, which was the native language of all the participants. Subsequently two independent individuals fluent in both Sinhalese and English translated the transcribed data to English. The independent translations were compared with the research team with the help of the independent translators for accuracy of content. A final document was then prepared after consensus had been reached on the translated transcript contents. Data were analyzed using a framework approach[13]. This involved the researchers reading through the transcripts and developing a matrix of overarching and supporting themes. A framework approach provides an effective route map for the research process and facilities both a case and theme based approach to data analysis[13].

## Results

### Patients

Over a four-month period we recruited 40 patients with type 2 diabetes, comprising 11 (28.2%) males and 29 (71.7%) females. Five of the selected 45 patients declined to participate or did not participate in the interviews. The mean age of the population was 55.4 (SD 8.9) years. The males (mean 56.4 years) were slightly older than the females (55.03 years). The patients were generally from a poor socio economic background with low levels of education, high unemployment and low incomes. Seventy one percent of the study population had not completed secondary education and 55% were never employed. The mean monthly income was less than 100USD per person. The mean BMI was 25.8kg/m2. The females had higher BMI than the males (males: 24.2 (SD 3.4), females: 26.67 (SD 4.2) p<0.001).

The mean duration of diabetes in the study population was 8.5 (SD 6.8) years. Over half the population (51.5%) had at least one diabetes related macro-vascular or micro-vascular complication.

### Theme 1: Engaging in physical activity

All participants admitted that they had received advice on the benefits of PA and regular exercise. Participants were invited to describe their PA. A common misconception noted among all the females and most of the males was the inability to distinguish a busy daily schedule from activities that promoted PA. All the females said they are very busy and engage in “work” from morning to night.

When encouraged to describe “physical activity” almost all the females described household chores such as cooking, doing the laundry, washing, caring for children/grandchildren and gardening. Few (n = 12, 33%) females described walking as PA but only 4 (10%) females walked for 30 minutes or more. Walking was mostly undertaken by females for visiting neighbouring houses of friends or relatives. The male participants engaged in more PA than females and were mostly for daily transportation needs such as getting to and from work. None of our participants had access to personalized transport such as cars or motorbikes, but 3 males used a non-motorized bicycle.

None of the participants engaged in a regular regimented exercise programme, and any significant PA performed was part of their lifestyle.

### Theme 2: Barriers to physical activity

Most participants (n = 38,95%)) mentioned that they encountered some form of barrier to engaging in regular PA. Three superordinate and 9 subordinate themes were developed following analysis with selected quotations from the transcripts. The barriers described by the participants were grouped into 3 themes; health related, time and lifestyle management and social. More than half the participants had more than one barrier to engaging in exercise.

#### Health related

A large number (n = 18, 45%) of participants said joint related problems prevented them from engaging in PA. The loose term “arthritis” was used to describe weight bearing-large joint problems. “Breathing problems” were described as another reason for not engaging in PA by 5(12.5%) participants. Few (n = 3, 7.5%) described “chest pain when exercising” as a barrier.

#### Time and lifestyle management

“Lack of time”, “inability to effectively manage time” and “lack of motivation” was highlighted by both males and females as barriers to engaging in an exercise programme. Few (n = 15,37.5%) described their daily chores, household activities and employment as barriers to engaging in PA.

#### Environmental, social and cultural

All the females stated they were embarrassed and “uncomfortable” to exercise in public areas. Most participants stated that they did not have access to a suitable facility for exercising. A majority stated that PA outside their daily activities was alien to their lifestyle. Few males (n = 5,12.5%) and all females were concerned on how exercising within the home or their closed community would be accepted by others. Majority recounted many instances of walking into puddles, stepping on to irregular roads and pavements and being chased by unrestrained animals such as dogs and cattle as significant events that limited regimented exercise.

### Theme 3: Overcoming barriers to exercise

Availability of privacy to engage in exercise was felt to be essential among most females. Both males and females felt that the availability of equipment and dedicated areas for exercise would improve PA. Most females felt that more support from family members to relieve them of their busy household schedules would encourage PA through regular exercise.

## Discussion

To our knowledge this study is the first qualitative study to exclusively explore the barriers to PA and exercise among Sri Lankan type 2 diabetic patients. The study population had a high BMI and high waist circumference. The population was middle aged to elderly, predominantly retired or unemployed, with a low income and lower level of education. The sample was from a centre having a catchment of semi-urban and rural dwellers.

PA is known to both prevent and control diabetes independently as well as through weight control [14]. In a study performed in the UK, South Asians were found to be less active compared to other ethnic minorities[15]. However, this and previous studies used measurements of PA that are in alignment with a Western life style. In Sri Lanka, the amount of PA performed during daily chores, remains largely unaccounted for when Euro-centric measures are used to quantify PA. While this may cause underreporting of PA, there is no other validated tool to measure PA of communities that demand significant physical exertion as a part of daily living.

Furthermore, at present there are no studies from the South Asian region that explore the barriers for PA and exercise among rural communities living in their endogenous environment. Therefore the present study remains unique.

In the current study, the participants were aware of the importance and benefits of PA. However, their knowledge was vague about the type and duration of PA. Interestingly, most of the female participants had difficulty in distinguishing a busy schedule from a physically active lifestyle. Walking was the commonest PA undertaken among our participants. However this was both irregular and variable, and was mostly performed on social and transportation requirements rather than a health promoting activity. A recent qualitative study performed in Sri Lanka had similar findings regarding PA among diabetic patients [16]. The inability to distinguish PA from daily activities has been reported previously from women of Asian origin [17].

In many Asian communities including Sri Lanka, the female gender has always been at a disadvantage due to many ethno-cultural factors. Important family decisions are most often made by the males of the family, often leading to large proportion of indoor household chores being shouldered by the female, thus leaving her with inadequate time to attend to her wellbeing. [18]

Almost all the participants said that they had barriers preventing them from engaging in PA. “Joint related issues” and “breathing problems” emerged as a limiting health issue. “Inability to prioritize time”, “lack of motivation” and “inability to find time for PA” due to household chores or employment” were the commonest time and lifestyle related causes. Socially, embarrassment, prioritizing of domestic activities and uncertainty on the social acceptance regimented exercise were common reasons for not engaging in PA.

Fear of engaging in PA in the presence of diabetes and other comorbidities has previously been reported in Sri Lanka and elsewhere [16,19,20]. Similarly lack of motivation and inability to prioritize time has been reported as significant barriers to exercise in previous studies[21,22]. The female participants in particular noted that they would be too embarrassed to undertake exercise in public areas as they felt this was culturally alien to them. This finding is in alignment with previously reported research performed in Asian ethnic minorities living in the UK and the USA [15,17,23].

Similarly, family and social pressures to prioritize domestic responsibilities over PA among women of Asian origin has been previously reported as barrier to PA[17,20,24].

The current study is unique in that it was performed on a population of rural, low income, predominantly middle aged to elderly population from a low resource setting. Previous studies originating on Asians and PA have originated from ethnic minority communities living in affluent societies [15,17,24,25]. A recent meta-analysis has also confirmed the lack of data on PA, of patients living in their indigenous settings[25].

We recommend that patient education regarding PA and exercise be more specific leaving little or no ambiguity regarding the type and duration of PA. We also recommend that where PA can be part of the daily lifestyle, the patients and health care givers be educated to acknowledge and encourage it. More robust and culturally accepted methods of knowledge transfer should be explored, such as the existing primary health care system. At the same time, PA prescription should be more individualized taking into account the physical disabilities and perceived negative outcomes and fears. Cultural and social beliefs and traditions should be taken into account in the formulation of PA activity guidelines at community and national levels. Development of infrastructure for exercise should be aligned with cultural beliefs and social norms, in an attempt to incorporate PA into daily lifestyles such as walking. Empowering of rural communities and community leaders with the knowledge and benefits of PA may help it to be more widely accepted among the elderly, less educated and the female gender.

### Strengths

This study is valuable in highlighting barriers encountered by a rural Asian population living in a resource poor environment. The qualitative design, supported by individual in-depth interviews was helpful in exploring contextual data not previously elicited by other approaches. The individual interviews were held in native Sinhalese thus facilitating a greater length of exploring in a population of elderly participants with limited education.

### Limitations

Limitations of this study may include selection bias and potential contamination bias in view of the purposeful sampling performed by us. The participants were recruited and interviewed at a clinic setting rather than in their own community, which may have limited the discourse of some participants.

## Conclusion

Our study revealed several recurring themes on PA patterns and barriers to PA in a rural community. Lack of uniform health education, health, lifestyle and social barriers were highlighted in this study. Culturally appropriate programmes that strengthen health education and empowering communities to overcome socio-economic barriers that limit PA should be implemented to better manage diabetes among rural Sri Lankan diabetic patients. We further highlight that new tools need to be developed that take into account PA of daily living when rural endogenous populations are studied.
